# A Note on Stronger Forms of Sensitivity for Non-Autonomous Dynamical Systems on Uniform Spaces

**DOI:** 10.3390/e26010047

**Published:** 2024-01-02

**Authors:** Lixin Jiao, Heyong Wang, Lidong Wang, Nan Wang

**Affiliations:** 1 Department of Electronic Business, South China University of Technology, Guangzhou 510006, China; jlxchaos@scut.edu.cn; 2 School of Disciplinary Basics and Applied Statistics, Zhuhai College of Science and Technology (Zhuhai College of Jilin University), Zhuhai 519041, China; wld@zcst.edu.cn; 3 School of Mathematics, Jilin University, Changchun 130012, China; wangnanchaos@126.com

**Keywords:** non-autonomous dynamical system, uniform space, multi-sensitivity with respect to a vector

## Abstract

This paper introduces the notion of multi-sensitivity with respect to a vector within the context of non-autonomous dynamical systems on uniform spaces and provides insightful results regarding N-sensitivity and strongly multi-sensitivity, along with their behaviors under various conditions. The main results established are as follows: (1) For a k-periodic nonautonomous dynamical system on a Hausdorff uniform space (S,U), the system $\left(S,f_{k}\circ \cdots \circ f_{1}\right)$ exhibits N-sensitivity (or strongly multi-sensitivity) if and only if the system $\left(S,f_{1,\infty }\right)$ displays N-sensitivity (or strongly multi-sensitivity). (2) Consider a finitely generated family of surjective maps on uniform space (S,U). If the system $\left(S,f_{1,\infty }\right)$ is N-sensitive, then the system $\left(S,f_{k,\infty }\right)$ is also N-sensitive. When the family $f_{1,\infty }$ is feebly open, the converse statement holds true as well. (3) Within a finitely generated family on uniform space (S,U), N-sensitivity (and strongly multi-sensitivity) persists under iteration. (4) We present a sufficient condition under which an nonautonomous dynamical system on infinite Hausdorff uniform space demonstrates N-sensitivity.

## 1. Introduction

Chaos refers to the inherent unpredictability that arises in deterministic systems in the absence of stochastic variables. It is a fundamental area of study in nonlinear science, representing a universal dynamical behavior of nonlinear systems. Furthermore, chaos profoundly and globally influences the evolution of nonlinear dynamics. Sensitivity is a critical element of chaos, attracting significant attention from scholars for research purposes [1,2,3,4,5,6,7,8,9,10,11,12,13,14].

Ruelle and Takens provided the first definition of sensitivity in 1971 [1]. It describes the unpredictable nature of chaotic processes and is essential to different kinds of chaos. Even a small change in a dynamical system’s initial configuration might result in significantly different behavior later on. In 1980, Auslander and Yorke applied sensitivity to topological dynamical system [2]. In 1989, the famous Devaney chaos was proposed [3]. Since then, the study of sensitivity became popular. If a system is topologically transitive, contains a dense collection of periodic points, and is sensitive to initial conditions, it is said to be Devaney chaotic [3]. Later, Banks et al. proved that the third condition (sensitivity) of the Devaney chaos is implied by the first two characteristics (transitivity and dense periodic points set) [4]. Glasner and Weiss expanded it to demonstrate that a transitive non-minimal system with dense minimal points is sensitive [5].

One indicator of a system’s sensitivity is the “largeness” of the time set where sensitivity occurs. From this perspective, Moothathu presented a number of stronger forms of sensitivity, namely, cofinite sensitivity, multisensitivity, and syndetic sensitivity [6]. His work further deepened the study of sensitivity. Later, Li presented the concept of ergodic sensitivity [7], which is another stronger form of sensitivity. He also present some sufficient conditions for dynamical system (X,f) to be ergodically sensitive. Liu et al. introduced thick sensitivity, syndetic sensitivity, thickly syndetic sensitivity, and strong sensitivity [8]. For more results about sensitivity, we refer to [9,10,11,12,13,14]

Recently, there has been significant research interest in studying sensitivity and chaoticity on uniform spaces for dynamical systems. Ahmadi et al. studied the topological shadowing property, chain transitivity, total chain transitivity, and chain mixing property for dynamical systems on uniform spaces [15]. Shah et al. presented and investigated the concept of distributional chaos on uniform spaces [16]. The notions of weak uniformity, uniform rigidity, and multi-sensitivity for uniform spaces were initially proposed by Wu et al. in 2019 [17]. The concepts of mean sensitivity and Banach mean sensitivity are expanded to uniform spaces by Wu et al. [18].

Consider a continuous map f:X→X operating on a compact metric space (X,d). A non-autonomous discrete system difference equation refers to

(1) $$x_{n+1}=f_{n}\left(x_{n}\right),n\geq 1.$$

where each $f_{n}$ is a continuous self-map on *X*. It should be noted that the autonomous dynamical system is a particular case of system (1). For other concepts and notations covered in this section, see Section 2.

The study of non-autonomous dynamical systems focuses on systems that vary with time, and compared to autonomous dynamical systems, the dynamics of non-autonomous dynamical systems are more complex. The applications of non-autonomous dynamical systems have been explored in various fields, including ecology, economics, climate science, biomedicine, and control engineering. These systems offer a valuable tool for understanding and predicting the behavior of dynamic systems under external influences and disturbances. By considering the time-varying nature of these systems, researchers can gain deeper insights into the intricacies of their dynamics and make more accurate predictions. The versatility and wide-ranging applications of non-autonomous dynamical systems make them an essential framework for studying and analyzing complex real-world phenomena. As a result, academics have been drawn to examine the complexity of such systems in recent years due to the rich dynamics [19,20,21,22,23,24,25,26,27].

Salman et al. introduced the notions of sensitivity, multi-sensitivity, cofinite sensitivity, and syndetic sensitivity within the realm of nonautonomous dynamical systems on uniform spaces. Additionally, they identified several adequate conditions wherein topological transitivity and the presence of densely distributed periodic points lead to sensitivity in nonautonomous systems residing on Hausdorff uniform spaces [28].

Inspired by their works, we present the notions of multi-sensitivity with respect to a vector, as well as N-sensitivity and strongly multi-sensitivity, within the context of nonautonomous dynamical systems on uniform spaces. Furthermore, we provide criteria under which a nonautonomous dynamical system on infinite Hausdorff uniform space demonstrates N-sensitivity.

The rest of the paper is organized as follows. In Section 2, some basic concepts are given. In Section 3, some stronger versions of sensitivities, namely, multi-sensitivity with respect to a vector, N-sensitive and strongly multi-sensitivity are introducd to nonautonomous dynamical systems on uniform spaces. In Section 4, it gives some sufficient conditions for an infinite Hausdorff uniform space to be N-sensitive.

## 2. Preliminaries

Throughout the paper, consider the symbols N={1,2,…} and $Z^{+}=\left\{0,1,\ldots \right\}$. Consider a nonempty set *S*, the diagonal of S×S is denoted by $\Delta_{S}=\left\{\left(x,x\right):x\in S\right\}$. Suppose that A⊂S×S, $A^{-1}$ which is defined by $A^{-1}=\left\{\left(y,x\right):\left(x,y\right)\in A\right\}$ is called the inverse of *A*. Specially, if $A=A^{-1}$, then *A* is said to be symmetric. Assume that $A_{1},A_{2}$ are subsets of S×S, the composite $A_{1}\circ A_{2}$ refer to the collection $\left\{\left(x,y\right)\in S\times S:\;\mathrm{there}\mathrm{exits}\;z\in S\;\mathrm{such}\mathrm{that}\;\left(x,z\right)\in A_{1}\;\mathrm{and}\;\left(z,y\right)\in A_{2}\right\}$. Denote $D_{S}=\left\{A\subset S\times S:\Delta_{S}\subset A\;\mathrm{and}\;A=A^{-1}\right\}$.

The introduction of the notion of uniform space was attributed to Weil in [29]. In this section, we provide a concise overview of uniform space, but for a more in-depth understanding, readers are encouraged to refer to ([30], Chapter 8) for a comprehensive introduction to the subject.

**Definition** **1** ([31])**.** *Let U be a nonempty sets consist of the subsets of S×S, U is called a uniform structure on S, if the following conditions hold:*

1. *$U\subset D_{S}$;*
2. *If $A_{1}\in U$ and $A_{1}\subset A_{2}\in D_{s}$, then $A_{2}\in U$;*
3. *For any $A_{1},A_{2}\in U,A_{1}\cap A_{2}\in U$;*
4. *For any A∈U, there exists B∈U such that B∘B⊂A.*

A uniform space is defined as a pair (S,U), where *S* is a non-empty set and U is a uniform structure on it. Generally, we call U entourages.

In a uniform space denoted as (S,U), a uniform topology can be established on *S*. This is characterized by a neighborhood base at each point *s* that belongs to *S*. This neighborhood base is composed of sets D[s]={t∈S:(s,t)∈D}, with *D* representing all entourages of the uniform space (S,U). For a map f:S→S on a uniform space (S,U), if ${\left(f\times f\right)}^{-1}\left(U\right)\subset U$, then *f* is said to be uniformly continuous on (S,U).

Next, we will introduce non-autonomous dynamical systems on uniform spaces. A nonautonomous discrete system is defined as $\left(S,f_{1,\infty }\right)$, (S,U) is a nontrivial uniform space and for any n∈N, $f_{n}:S\to S$ is uniformly continuous. This system consists of a sequence of uniformly continuous maps $f_{1,\infty }={\left\{f_{n}\right\}}_{n=1}^{\infty }$ acting on the uniform space *S*. Denote that $O\left(x\right)=\{f_{1}^{n}\left(x\right):n\geq 0\}$ is the orbit of *x* for each x∈S, where $f_{1}^{n}=f_{n}\circ \cdots \circ f_{1}\left(n\geq 1\right)$ and $f_{1}^{\left(0\right)}=id$. When $f_{n}=f$ for any n∈N, the above system is an autonomous dynamical system (S,f). For i,n∈N, let $f_{i}^{n}:=f_{i+n-1}\circ f_{i+n-2}\circ \cdots \circ f_{i},f_{i}^{\left(0\right)}:=id$ and the kth iterate $f_{1,\infty }^{\left[k\right]}={\left\{f_{k(n-1)+1}^{k}\right\}}_{n=1}^{\infty }$.

Consider a nonautonomous dynamical system $\left(S,f_{1,\infty }\right)$, if $\left\{f_{n}:n\in N\right\}$ is a finite set, $\left(S,f_{1,\infty }\right)$ is called finitely generated. If there is an l∈N satisfing $f_{n+lm}=f_{n}$ for each m∈N and each 1≤n≤l, $\left(S,f_{1,\infty }\right)$ is said to be periodic. $\left(S,f_{1,\infty }\right)$ is feebly open if for each nonempty set U⊂S and each n∈N, $int(f_{n}\left(U\right))\neq \emptyset$ (where int(V) denote the interior of the set *V*).

For the periodic points for non-autonomous dynamical systems, scholars proposed two different definitions from different perspectives. Here, the two different periodic points is distinguish by P1 and P2.

**Definition** **2** ([27,32])**.** *(P1) Consider a point x in S, x is called P1-periodic, if there is an n∈N such that $f_{1}^{ni}\left(x\right)=x$ for each i∈N.*

*(P2) Consider a point x of S, x is called P2-periodic, if there is an n∈N such that $f_{1}^{\left(i+n\right)}\left(x\right)=f_{1}^{n}\left(x\right)$ for each i≥0.*

Obviously, P2-periodic implies P1-periodic and the orbit of a periodic point in the sense of (P2) must be finite. However, by the Example 4.4 of [33], the orbit of a periodic point in sense of (P1) has the potential to be infinite.

Assume that *D* is a subset of S×S and *V* is a subset of *S*. Denote

$$N_{f_{1,\infty }}\left(V,D\right)=\left\{n\in N:\;\mathrm{there}\mathrm{exist}\;x,y\in V\;\mathrm{such}\mathrm{that}\;\left(f_{1}^{\left(n\right)}\left(x\right),f_{1}^{\left(n\right)}\left(y\right)\right)\notin D\right\}.$$

Assume that P is the collection of all subsets of $Z^{+}$. F⊂P is called a Furstenberg family if it satisfies $F_{1}\subset F_{2}$ and $F_{1}\in F$ imply $F_{2}\in F$ (for details see [34]).

The following definition about sensitivity were generated by Huang et al. [35] and Salman et al. [28]. Let F be a Furstenberg family. A non-autonomous dynamical system $\left(S,f_{1,\infty }\right)$ on a uniform space (S,U) is said to be sensitive, if there exists an entourage E∈U such that for any nonempty open subset *V* of *S*, $N_{f_{1,\infty }}\left(V,E\right)\neq \emptyset$. Typically, *E* is called a sensitive entourage. If $N_{f_{1,\infty }}\left(V,E\right)$ is cofinite set, i.e., $N/N_{f_{1,\infty }}\left(V,E\right)$ is finite, then the system becomes cofinitely sensitive. The entourage is called cofinitely sensitive entourage respectively. when $N_{f_{1,\infty }}\left(V,E\right)$ is syndetic set, i.e., there exists N∈N such that $\left\{i,i+1,\ldots ,i+N\right\}\cap N_{f_{1,\infty }}\left(V,E\right)\neq \emptyset$ for every $i\in Z^{+}$, the system is called syndetically sensitive. If $N_{f_{1,\infty }}\left(V,E\right)\in F$, the system is said to be F-sensitive. If there is an entourage E∈U such that for each k∈N and each nonempty open subsets $V_{1},\ldots ,V_{k}$ of *S*, ${⋂}_{i=1}^{k}N_{f_{1,\infty }}\left(V_{i},E\right)\neq \emptyset$, then the system is called multi-sensitive. The entourage *E* is called multi-sensitive entourage, respectively.

**Definition** **3** ([36])**.** *A system $\left(S,f_{1,\infty }\right)$ is said to be multi-transitive if $f_{1,\infty }^{\left[1\right]}\times f_{1,\infty }^{\left[2\right]}\times \cdots \times f_{1,\infty }^{\left[m\right]}:S^{m}\to S^{m}$ is topologically transitive for every m∈N. Equivalently, if for every collection of nonempty open subsets $U_{1},U_{2},\cdots ,U_{m};V_{1},V_{2},\cdots ,V_{m}$ of S, there exists an l∈N such that $f_{1}^{jl}\left(U_{j}\right)\cap V_{j}\neq \emptyset$, for each j∈{1,2,…,m} and for every m∈N.*

## 3. Multi-Sensitivity with Respect to a Vector for Nonautonomous Dynamical System on Uniform Spaces

This section will introduce some stronger versions of sensitivity, namely, multi-sensitivity with respect to a vector, N-sensitive and strongly multi-sensitivity for nonautonomous dynamical systems on uniform spaces.

**Definition** **4.** *Consider a nonautonomous dynamical system $\left(S,f_{1,\infty }\right)$, where (S,U) is a uniform space. Let $\vec{a}=\left(a_{1},a_{2},\ldots a_{r}\right)$. (S,U) is said to be*

1. *multi-sensitive with respect to $\vec{a}$, if there exists an entourage D∈U such that for any nonempty open subsets $U_{1},U_{2},\cdots U_{r}$ of S, $\overset{r}{\underset{i=1}{⋂}}N_{f_{1,\infty }^{\left[a_{i}\right]}}\left(U_{i},D\right)\neq \emptyset$.*
2. *N-sensitive, if there exists an entourage D∈U, for any $U_{1},U_{2},\cdots U_{r}\subset S$, $\overset{r}{\underset{i=1}{⋂}}N_{f_{1,\infty }^{\left[i\right]}}$$(U_{i},D)\neq \emptyset$.*
3. *strongly multi-sensitive, if there is an entourage D∈U, $\left(S,f_{1,\infty }\right)$ is multi-sensitive with respect to any vector in $N^{n}$ and any n∈N.*

Clearly, cofinite sensitive ⇒ strongly multi-sensitive ⇒ multi-sensitive

strongly multi-sensitive ⇒N-sensitive

**Theorem** **1.** *A k-periodic nonautonomous dynamical system $\left(S,f_{1,\infty }\right)$ on a Hausdorff uniform space is N-sensitive if and only if $\left(S,f_{k}\circ \cdots \circ f_{1}\right)$ is as well.*

**Proof.**  For convenience, denote $g=f_{k}\circ \cdots \circ f_{1}$

“⇒”. Let $U_{1},U_{2},\cdots U_{m}$ be nonempty open sets of *S*. Assume the (S,g) is N-sensitive with respect to a entourage D∈U. Suppose that $n\in \overset{m}{\underset{i=1}{⋂}}N_{g^{i}}\left(U_{i},D\right)\neq \emptyset$ and $\left(S,f_{1,\infty }\right)$ is k-periodic. then $kn\in \overset{m}{\underset{i=1}{⋂}}N_{f_{1,\infty }^{\left[i\right]}}\left(U_{i},D\right)\neq \emptyset .$

“⇐”. Let $\left(S,f_{1,\infty }\right)$ be N-sensitive with respect to D∈U. For any non-empty open sets $U_{1},U_{2},\ldots U_{m}$, let $U_{i}^{\prime }=U_{1},U_{k+i}^{\prime }=U_{2},\ldots U_{(m-1)k+i}^{\prime }=U_{m}$, for any 1≤i≤k.

As $\left(S,f_{1,\infty }\right)$ is N-sensitive, $\overset{km}{\underset{i=1}{⋂}}N_{f_{1,\infty }^{\left[i\right]}}\left(U_{i}^{\prime },D\right)\neq \emptyset .$ Take $n\in \overset{km}{\underset{i=1}{⋂}}N_{f_{1,\infty }^{\left[i\right]}}\left(U_{i}^{\prime },D\right)$, using k periodicity of $\left(S,f_{1,\infty }\right)$, $n\in \overset{m}{\underset{i=1}{⋂}}N_{{\left(f_{1,\infty }^{k}\right)}^{\left[i\right]}}\left(U_{i},D\right)=\overset{n}{\underset{i=1}{⋂}}N_{g^{\left[i\right]}}\left(U_{i},D\right)$.

Therefore, $\overset{n}{\underset{i=1}{⋂}}N_{g^{\left[i\right]}}\left(U_{i},D\right)\neq \emptyset .$ Thus, (S,g) is N-sensitive. □

**Theorem** **2.** *A k-periodic nonautonomous dynamical system $\left(S,f_{1,\infty }\right)$ on Hausdorff uniform space is strongly multi-sensitive if and only if $\left(S,f_{k}\circ \cdots \circ f_{1}\right)$ is as well.*

**Proof.**  For convenience, denote $g=f_{k}\circ \cdots \circ f_{1}$. Note that as $\left(S,f_{1,\infty }\right)$ is k-periodic, ${\left(f_{1}^{k}\right)}^{s}=f_{1}^{ks}$.

For the necessity, assume that (S,g) is strongly multi-sensitive, where $g=f_{k}\circ f_{k-1}\circ \cdots \circ f_{1}$. For any vector $\vec{a}=\left(a_{1},a_{2},\ldots ,a_{m}\right),m\in N$ and any non-empty open sets $U_{1},U_{2},\ldots ,U_{m}$, as (S,g) is multi-sensitive with respect to $\vec{a}$, that is, there exists an entourage D∈U such that $\overset{m}{\underset{i=1}{⋂}}N_{g^{\left[a_{i}\right]}}\left(U_{i},D\right)\neq \emptyset$. As system $\left(S,f_{1,\infty }\right)$ is k-periodic, so $\overset{m}{\underset{i=1}{⋂}}N_{g^{\left[i\right]}}\left(U_{i},D\right)=\overset{m}{\underset{i=1}{⋂}}N_{{\left(f_{1}^{k}\right)}^{\left[a_{i}\right]}}\left(U_{i},D\right)=\overset{m}{\underset{i=1}{⋂}}N_{f_{1}^{\left[ka_{i}\right]}}\left(U_{i},D\right)\neq \emptyset$, hence $\overset{m}{\underset{i=1}{⋂}}N_{f_{1}^{\left[a_{i}\right]}}\left(U_{i},D\right)\neq \emptyset$.

For the sufficiency, suppose that $\left(S,f_{1,\infty }\right)$ is strongly multi-sensitive. For any m∈N and any vector $\vec{a}=\left(a_{1},a_{2},\ldots ,a_{m}\right)$, let $U_{1},U_{2},\ldots ,U_{m}$ be any nonempty open sets of *S*. By the hypothesis, $\left(S,f_{1,\infty }\right)$ is multi-sensitive with respect to vector $\left(ka_{1},ka_{2},\ldots ,ka_{m}\right)$. That is, there is an entourage *D* in U such that $\overset{m}{\underset{i=1}{⋂}}N_{f_{1,\infty }^{\left[ka_{i}\right]}}\left(U_{i},D\right)\neq \emptyset$. As $f_{1}^{ks}={\left(f_{1}^{k}\right)}^{s}$ for, we can get $\overset{m}{\underset{i=1}{⋂}}N_{{\left(f_{1}^{k}\right)}^{\left[a_{i}\right]}}\left(U_{i},D\right)\neq \emptyset$. Hence $\overset{m}{\underset{i=1}{⋂}}N_{g^{\left[a_{i}\right]}}\left(U_{i},D\right)\neq \emptyset$.

□

**Theorem** **3.** *A nonautonomous dynamical system $\left(S,f_{1,\infty }\right)$ on uniform space (S,U) is N-sensitive if and only if for any nonempty open sets $U_{1},U_{2},\ldots U_{m}$, $\overset{m}{\underset{i=1}{⋂}}N_{f_{1,\infty }^{\left[i\right]}}\left(U_{i},D\right)\in F_{inf}$, where $F_{inf}$ denote the collection of all infinite subsets of $Z^{+}$.*

**Proof.**  Since sufficiency is obvious, we only need to prove necessity. Assume that (S,U) is N-sensitive with sensitive entourage D∈U. We use the counterfactual. Suppose $U_{1},U_{2},\cdots U_{m}$ are any nonempty open subset of *S* and $\overset{m}{\underset{i=1}{⋂}}N_{f_{1,\infty }^{\left[i\right]}}\left(U_{i},D\right)\notin F_{inf}$. Denote *k* is the maximum of the set $\overset{m}{\underset{i=1}{⋂}}N_{f_{1,\infty }^{\left[i\right]}}\left(U_{i},D\right)$ and take $\hat{U}=D\cap \underset{1\leq n\leq mk}{⋂}{\left(f_{n}\circ f_{n-1}\circ \ldots \circ f_{1}\right)}^{2}\left(D\right)$. Given *S* is a uniform space, there exists an $\hat{V}\in U$ such that $\hat{V}\circ \hat{V}\subset \hat{U}$. Fix $z_{i}\in U_{i}$ and let $U_{i}^{\prime }=U_{i}\cap \hat{V}\left(z_{i}\right),1\leq i\leq m$. Choose open neighhoors $W_{i}$ of $z_{i}$ with $W_{i}\subset U_{i}^{\prime },1\leq i\leq m$. Clearly, for any $x_{i},y_{i}\in W_{i}$, $\left(x_{i},y_{i}\right)\in \hat{V}\circ \hat{V}\subset \hat{U}$. This indicates that for any given 1≤n≤mk, $\left(f_{1}^{\left(n\right)}\left(x_{i}\right),\;f_{1}^{\left(n\right)}\left(y_{i}\right)\right)=\left(f_{n}\circ \cdots \circ f_{1}\left(x_{i}\right),\;f_{n}\circ \cdots \circ f_{1}\left(y_{i}\right)\right)\in D$. According to the N-sensitivity of $f_{1,\infty }$, it can be deduce the existence of *ℓ* and $x_{i}^{\prime },y_{i}^{\prime }\in U_{i}$ such that $(f_{1}^{i\ell }\left(x_{i}^{\prime }\right),\;f_{1}^{i\ell }\left(y_{i}^{\prime }\right))\notin D$. This means ℓ>mk>k. This contradicts with the notion that *k* is the maximum of the set $\overset{m}{\underset{i=1}{⋂}}N_{f_{1,\infty }^{\left[i\right]}}\left(U_{i},D\right)$. □

**Remark** **1.** *By employing analogous reasoning, it can be confirmed that the aforementioned theorem holds true for cases of strong multi-sensitivity as well.*

We subsequently demonstrate that for a modified nonautonomous dynamical system $\left(S,f_{k,\infty }\right)$ with a feeble open family $f_{1,\infty }$, where $\left(S,f_{k,\infty }\right)={\left\{f_{n}\right\}}_{n=k}^{\infty }$, N-sensitivity as well as strongly multi-sensitivity are preserved.

**Theorem** **4.** *Assume that (S,U) is a uniform space and $f_{1,\infty }$ is finitely generated, where each $f_{i}$ be surjective. If the system $\left(S,f_{1,\infty }\right)$ exhibits N-sensitivity, it follows that the system $\left(S,f_{k,\infty }\right)$ also displays N-sensitivity. Conversely, if the family $f_{1,\infty }$ is feebly open, the converse holds true as well.*

**Proof.**  As $\left(S,f_{1,\infty }\right)$ is N-sensitive and by Theorem 3, there exists an entourage D∈U such that for any nonempty open sets $U_{1},U_{2},\cdots ,U_{m}\subset S$, $\overset{m}{\underset{i=1}{⋂}}N_{f_{1,\infty }^{\left[i\right]}}\left(U_{i},D\right)\in F_{inf}$.

Consider a set of generators $g_{1},g_{2},\cdots g_{s}$ for the $f_{1,\infty }$, and let’s fix an arbitrary integer *k* greater than or equal to 2. Define $\Gamma :=\underset{1\leq n\leq 2mk}{⋃}{\left\{1,2,\cdots ,s\right\}}^{n}$, then take any element α from the set Γ, denote $\alpha =(\alpha_{1},\alpha_{2},\cdots ,\alpha_{\ell })$ for 1≤n≤2mk. We define the composition of these generators as $g_{\alpha_{\ell }}\circ g_{\alpha_{\ell -1}}\circ \cdots \circ g_{\alpha_{1}}=g_{\alpha }$.

Clearly, each map $g_{\alpha }$, where α∈Γ, exhibits uniform continuity. This observation leads us to conclude that the set $E=D\cap \underset{\alpha \in \Gamma }{⋂}{\left(g_{\alpha }\times g_{\alpha }\right)}^{-1}\left(D\right)\in U$.

As $f_{1}^{\left(k-1\right)}$ is surjective and continuous, it follows that $V_{i}={\left(f_{1}^{\left(k-1\right)}\right)}^{-1}\left(U_{i}\right),\left(i=1,2,\cdots ,m\right)$ are nonempty open sets, by the N-sensitivity of $f_{1,\infty }$ and Theorem 3, $\overset{m}{\underset{i=1}{⋂}}N_{f_{1,\infty }^{\left[i\right]}}\left(V_{i},D\right)\in F_{inf}$, there exists $x_{i}^{\prime },y_{i}^{\prime }\in V_{i}$ and M>2k such that $(f_{1}^{\left(iM\right)}\left(x_{i}^{\prime }\right),f_{1}^{\left(iM\right)}\left(y_{i}^{\prime }\right))\notin D$. Denote that $x_{i}=f_{1}^{\left(k-1\right)}\left(x_{i}^{\prime }\right),y_{i}=f_{1}^{\left(k-1\right)}\left(y_{i}^{\prime }\right),i=1,2,\ldots ,m$. Obviously, for each i∈{1,2,…,m}, $x_{i},y_{i}\in U_{i}.$

Note that

$$\begin{array}{cc} \; & \left(f_{1}^{\left(iM\right)}\left(x_{i}^{\prime }\right),f_{1}^{\left(iM\right)}\left(y_{i}^{\prime }\right)\right)\\ = & \left(f_{iM}\circ f_{iM-1}\circ \cdots f_{k}\left(f_{1}^{\left(k-1\right)}\left(x_{i}^{\prime }\right)\right),f_{iM}\circ f_{iM-1}\circ \cdots \circ f_{k}\left(f_{1}^{\left(k-1\right)}\left(y_{i}^{\prime }\right)\right)\right)\\ = & \left(f_{iM}\circ \cdots \circ f_{k}\left(x_{i}\right),f_{iM}\circ \cdots \circ f_{k}\left(y_{i}\right)\right)\\ = & (f_{k}^{iM-k+1}\left(x_{i}\right),f_{k}^{iM-k+1}\left(y_{i}\right))\notin D. \end{array}$$

Select a positive integer *p* and a non-negative integer *r* such that 0≤r≤k−1 and the equation M=pk+r holds. note that p≥2.

Denote that $\alpha^{i}=\left(\left(ip-i+1\right)k,\left(ip-i+1\right)k+1,\cdots ,iM\right)\left(i=1,2,\cdots ,m\right)$, as iM−(ip−i+1)k+1=ir+ik−k+1≤(2i−1)k+1≤2ik≤2mk(i=1,2,⋯,m), then for each i∈{1,2,…,m}, $\alpha^{i}\in \Gamma$ and $g_{\alpha^{i}}=f_{iM}\circ f_{iM-1}\circ \cdots \circ f_{(i(p-1)+1)k}$. This together with

$$\begin{array}{cc} \; & \left(f_{k}^{M-k+1}\left(x_{1}\right),f_{k}^{M-k+1}\left(y_{1}\right)\right)\\ \; & =(f_{M}\circ \cdots \circ f_{pk}\circ \left(f_{k}^{(p-1)k}\left(x_{1}\right)\right),f_{M}\circ \cdots \circ f_{pk}\circ \left(f_{k}^{(p-1)k}\left(y_{1}\right)\right))\\ \; & =(g_{\alpha^{1}}\left(f_{k}^{(p-1)k}\left(x_{1}\right)\right),g_{\alpha^{1}}\left(f_{k}^{(p-1)k}\left(y_{1}\right)\right))\notin D,\\ \; & \left(f_{k}^{2M-k+1}\left(x_{2}\right),f_{k}^{2M-k+1}\left(x_{2}\right)\right)\\ \; & =(f_{2M}\circ \cdots \circ f_{(2p-1)k}\circ \left(f_{k}^{2(p-1)k}\left(x_{2}\right)\right),f_{2M}\circ \cdots \circ f_{(2p-1)k}\circ \left(f_{k}^{2(p-1)k}\left(y_{2}\right)\right))\\ \; & =(g_{\alpha^{2}}\left(f_{k}^{2(p-1)k}\left(x_{2}\right)\right),g_{\alpha^{2}}\left(f_{k}^{2(p-1)k}\left(y_{2}\right)\right))\notin D.\\ \; & \vdots \\ \; & \left(f_{k}^{mM-k+1}\left(x_{m}\right),f_{k}^{mM-k+1}\left(y_{m}\right)\right)\\ \; & =(f_{mM}\circ \cdots \circ f_{(mp-m+1)k}\left(f_{k}^{m(p-1)k}\left(x_{m}\right)\right),f_{mM}\circ \cdots \circ f_{(mp-m+1)k}\left(f_{k}^{m(p-1)k}\left(y_{m}\right)\right))\\ \; & =(g_{\alpha^{m}}\left(f_{k}^{m(p-1)k}\left(x_{m}\right)\right),g_{\alpha^{m}}\left(f_{k}^{m(p-1)k}\left(y_{m}\right)\right))\notin D. \end{array}$$

By the choice of *E*, this implies that $(f_{k}^{i(p-1)k}\left(x_{i}\right),f_{k}^{i(p-1)k}\left(y_{i}\right))\notin E$, then $\left(p-1\right)k\in \overset{m}{\underset{i=1}{⋂}}N_{f_{k,\infty }^{\left[i\right]}}\left(U_{i},E\right)\neq \emptyset$.

Conversely, assume that $\left(S,f_{k,\infty }\right)$ is N-sensitive with sensitive entourage $D^{\prime }\in U$. For nonempty open sets $W_{1},W_{2},\cdots ,W_{m}$ of *S*, due to the feeble openness exhibited by the family $f_{1,\infty }$, it can be inferred that $W_{i}^{\prime }=int\left(f_{1}^{\left(k-1\right)}\left(W_{1}\right)\right)\left(1\leq i\leq m\right)$ are nonempty open subsets of *S*. As $\left(S,f_{k,\infty }\right)$ is N-sensitive, there exists $M\in \overset{m}{\underset{i=1}{⋂}}N_{f_{k,\infty }^{\left[i\right]}}\left(W_{i}^{\prime },D^{\prime }\right)\neq \emptyset$. This implies that there are $x_{i}^{\prime },y_{i}^{\prime }\in W_{i}^{\prime }$ such that $\left(f_{k}^{iM}\left(x_{i}^{\prime }\right),f_{k}^{iM}\left(y_{i}^{\prime }\right)\right)\notin D^{\prime }$.

Choose $x_{i},y_{i}\in W$ with $f_{1}^{\left(k-1\right)}\left(x_{i}\right)=x_{i}^{\prime }$ and $f_{1}^{\left(k-1\right)}\left(y_{i}\right)=y_{i}^{\prime }$. therefore

$$\begin{array}{cc} \; & \left(f_{1}^{iM+k-1}\left(x_{i}\right),f_{1}^{iM+k-1}\left(y_{i}\right)\right)\\ = & \left(f_{k}^{iM}\left(f_{1}^{\left(k-1\right)}\left(x_{i}\right)\right),f_{k}^{iM}\left(f_{1}^{\left(k-1\right)}\left(y_{i}\right)\right)\right)\\ = & \left(f_{k}^{iM}\left(x_{i}^{\prime }\right),f_{k}^{iM}\left(y_{i}^{\prime }\right)\right)\notin D^{\prime }. \end{array}$$

Denote that $\beta^{i}=\left(iM+1,iM+2,\ldots ,iM+k-2,iM+k-1\right)\in \Gamma$, therefore

(2) $$\begin{array}{cc} \; & \left(g_{\beta^{i}}\left(f_{1}^{iM}\left(x_{i}\right)\right),g_{\beta^{i}}\left(f_{1}^{iM}\left(y_{i}\right)\right)\right)\\ = & \left(f_{iM+k-1}\circ f_{iM+k-2}\circ \cdots \circ f_{iM+1}\left(f_{1}^{iM}\left(x_{i}\right)\right),f_{iM+k-1}\circ f_{iM+k-2}\circ \cdots \circ f_{iM+1}\left(f_{1}^{iM}\left(y_{i}\right)\right)\right)\\ = & \left(f_{1}^{iM+k-1}\left(x_{i}\right),f_{1}^{iM+k-1}\left(y_{i}\right)\right)\notin D^{\prime }. \end{array}$$

As each $g_{\beta }\left(\beta \in \Gamma \right)$ is uniform continuous, there exists $E^{\prime }=D^{\prime }\cap \underset{\beta \in \Gamma }{⋂}{\left(g_{\beta }\times g_{\beta }\right)}^{\left(-1\right)}\left(D^{\prime }\right)\in U$. This together with (3), implies that $\left(f_{1}^{iM}\left(x_{i}\right),f_{1}^{iM}\left(y_{i}\right)\right)\notin E^{\prime }\left(i=1,2,\cdots ,m\right)$, $M\in \overset{m}{\underset{i=1}{⋂}}N_{f_{1,\infty }^{\left[i\right]}}\left(W_{i},E^{\prime }\right)\neq \emptyset$. Therefore $\left(S,f_{1,\infty }\right)$ is N-sensitive. □

Evidently, the aforementioned outcome holds valid in the context of strong multi-sensitivity as well.

**Theorem** **5.** *Consider a finitely generated family $f_{1,\infty }$ on a uniform space (S,U). The pair $\left(S,f_{1,\infty }\right)$ possesses N-sensitivity if and only if, for any k≥1, the pair $\left(S,f_{1,\infty }^{\left[k\right]}\right)$ also exhibits N-sensitivity.*

**Proof.**  Sufficiency: This is readily evident.

Necessity: Consider a generator set $g_{1},g_{2},\cdots ,g_{s}$ for $f_{1,\infty }$, and let *k* be any integer greater than or equal to 2. Suppose that $f_{1,\infty }$ is N-sensitive with the N-sensitive entourage D∈U. For nonempty open sets $U_{1},U_{2},\ldots ,U_{m}\subset S$, according to the Theorem 3, $\overset{m}{\underset{i=1}{⋂}}N_{f_{1,\infty }^{\left[i\right]}}\left(U_{i},D\right)\in F_{inf}$. Consequently, there exist elements $x_{i}$ and $y_{i}$ in $U_{i}$, where ℓ>k, such that the pair $\left(f_{1}^{i\ell }\left(x_{i}\right),f_{1}^{i\ell }\left(y_{i}\right)\right)$ lies outside the bounds of *D*, that is $(f_{1}^{i\ell }\left(x_{i}\right),f_{1}^{i\ell }\left(y_{i}\right))\notin D$. Select a positive integer *p* and a non-negative integer *r* such that 0≤r≤k−1 and the equation M=pk+r holds. Denote $\Gamma :=\underset{1\leq n\leq m(k+1)}{⋃}{\left\{1,2,\ldots ,s\right\}}^{n}$, For any α∈Γ, where $\alpha =\left(\alpha_{1},\alpha_{2},\ldots ,\alpha_{\ell }\right),1\leq \ell \leq m\left(k+1\right)$, define $g_{\alpha_{\ell }}\circ \cdots \circ g_{\alpha_{1}}=g_{\alpha }$. Evidently, the uniform continuity holds for every $g_{\alpha }$ where α belongs to the set Γ. This leads to the implication that $E=D\cap \underset{\alpha \in \Gamma }{⋂}{\left(g_{\alpha }\times g_{\alpha }\right)}^{-1}\left(D\right)\in U$. Denote that $\alpha^{i}=(ipk+1,ipk+2,\ldots ,ipk+ir-1,$ipk+ir), i=1,2,…,m. Obviously, $\alpha^{i}\in \Gamma$. Hence

$$\begin{array}{cc} \; & \left(g_{\alpha^{i}}\left(f_{1}^{\left(ipk\right)}\left(x_{i}\right)\right),g_{\alpha^{i}}\left(f_{1}^{\left(ipk\right)}\left(y_{i}\right)\right)\right)\\ = & \left(f_{ipk+ir}\circ \cdots \circ f_{\left(ipk+1\right)}\left(f_{1}^{\left(ipk\right)}\left(x_{i}\right)\right),f_{ipk+ir}\circ \cdots \circ f_{\left(ipk+1\right)}\left(f_{1}^{\left(ipk\right)}\left(y_{i}\right)\right)\right)\\ = & \left(f_{1}^{i(pk+r)}\left(x_{i}\right),f_{1}^{i(pk+r)}\left(y_{i}\right)\right)\\ = & \left(f_{1}^{i\ell }\left(x_{i}\right),f_{1}^{i\ell }\left(y_{i}\right)\right)\notin D\left(i=1,2,\cdots ,m\right) \end{array}$$

Due to the selection of set *E*, it follows that $(f_{1}^{ipk}\left(x_{i}\right),f_{1}^{ipk}\left(y_{i}\right))\notin E$, i.e., $p\in \overset{m}{\underset{i=1}{⋂}}N_{f_{1,\infty }^{\left[ik\right]}}\left(U_{i},E\right)$, thus $\left(S,f_{1,\infty }^{\left[k\right]}\right)$ is N-sensitive. □

**Remark** **2.** *Using analogous reasoning, it can be established that the aforementioned theorem holds valid in the context of strong multi-sensitivity as well.*

## 4. Sufficient Condition for N-Sensitivity

In this section, We will present certain conditions that are adequate for an infinite Hausdorff uniform space to exhibit N-sensitivity.

**Definition** **5** ([28])**.** *For a sequence $f_{n}:S\to S$ within a uniform space (S,U), $f_{n}$ converges uniformly to f if for every entourage D in U, there exists a natural number N such that, for all x in S and all n≥N, the pair $(f_{n}\left(x\right),f\left(x\right))\in D$.*

**Lemma** **1** ([28] (Lemma 4.1))**.** *In a Hausdorff uniform space (S,U), a map f:S→S is uniformly continuous if and only if, for every D in U, there exists an entourage E in U such that, for each (x,y) of E, the pair (f(x),f(y))∈D.*

**Lemma** **2** ([28] (Proposition 4.1))**.** *Consider a Hausdorff uniform space (S,U). Assume that the sequence of maps $f_{n}:S\to S$ uniformly converges to f. If each individual map $f_{n}$ is uniformly continuous, it follows that the map f itself is uniformly continuous.*

**Lemma** **3** ([28] (Proposition 4.2))**.** *Consider a Hausdorff uniform space (S,U). If the sequence of maps $f_{n}:S\to S$ uniformly converges to f, then for each natural number k, the k-th iteration of $f_{n}$, denoted as $f_{n}^{\left(k\right)}$, also uniformly converges to the k-th power of f, denoted as $f^{k}$.*

**Lemma** **4** ([36] (Lemma 3.1))**.** *If $\left(S,f_{1,\infty }\right)$ is multi-transitive, then the set $\ell \in N:f_{1}^{i\ell }\left(U_{j}\right)\cap V_{j}\neq \emptyset$, for each j∈{1,2,⋯,m}, for any collection of nonempty open sets $U_{1},U_{2},\cdots ,U_{m}$; $V_{1},V_{2},\cdots ,V_{m}$ is infinite.*

**Lemma** **5** ([28] (Theorem 4.1 Claim 1))**.** *Consider an infinite Hausdorff uniform space (S,U) without isolated points, and suppose the convergence of $\left(S,f_{1,\infty }\right)$ to f is uniform. Assuming that (S,U) possesses a dense set of periodic points as defined by (P1), there exists an entourage E∈U such that, for any x∈S, there exists a periodic point p of f such that, for all positive integers n, the pair $\left(x,f^{n}\left(p\right)\right)$ falls outside the scope of E, denoted as $(x,f^{n}\left(p\right))\notin E$*

**Theorem** **6.** *Consider an infinite Hausdorff uniform space (S,U) without isolated points, where the convergence of $\left(S,f_{1,\infty }\right)$ to f is uniform. If (S,U) satisfies both multi-transitivity and possesses a dense set of periodic points as defined by (P1), then it exhibits N-sensitivity.*

**Proof.**  Consider an entourage E∈U derived from Lemma 5, and select an entourage *D* in U such that *D* composed with itself four times, i.e., D∘D∘D∘D, is contained within *E*. For any nonempty open sets $U_{1},U_{2},\cdots ,U_{m}$, we can find $x_{i}\in U_{i}\left(i=1,2,\cdots ,m\right)$. Since $\left(S,f_{1,\infty }\right)$ has a dense set of periodic points, there exist periodic points $p_{i}^{\prime }$ of $f_{1,\infty }$ such that $p_{i}^{\prime }\in U_{i}\cap D\left[x_{i}\right]\left(i=1,2,\cdots ,m\right)$. Suppose that the period of each $p_{i}^{\prime }$ is $k_{i}\left(i=1,2,\cdots ,m\right)$. By Lemma 5, there exist periodic points $p_{i}$ of *f* satisfying that for all $n\in Z^{+}$, $(x_{i},f^{n}\left(p_{i}\right))\notin E$.

Applying Lemmas 2 and 3, Consequently, we can deduce that $f^{j}$ exhibits uniform continuity for every 0≤j≤mk, where $k=k_{1}k_{2}\cdots k_{m}$. This implies that for each i∈{1,2,…,m}, there is a small enough open set $V_{i}$ with $p_{i}\in V_{i}$ such that for each $z_{i}\in V_{i}$ and 0≤j≤mk, the following equation hold:

(3) $$(f^{j}\left(p_{i}\right),f^{j}\left(z_{i}\right))\in D$$

By leveraging the multi-transitivity of $\left(S,f_{1,\infty }\right)$ along with the insights from Lemma 4, we can conclude that the set $n\in Z^{+}:f_{1}^{\left(in\right)}\left(U_{i}\cap D\left[x_{i}\right]\right)\cap V_{i}\neq \emptyset$ is of infinite size. This implies the existence of both 0≤r<k and an increasing sequence ${\left\{n_{\ell }\right\}}_{\ell \in N}$, such that

(4) $$\left\{kn_{\ell }+r:\ell \in N\right\}\subset \left\{n\in Z^{+}:f_{1}^{\left(in\right)}\left(U_{i}\cap D\left[x_{i}\right]\right)\cap V_{i}\neq \emptyset \right\}.$$

Applying Lemma 3, yield that $f_{i(kn_{\ell }+r)+1}^{i(k-r)}$ converges uniformly to $f^{i(k-r)}$ as ℓ→∞, i=1,2,⋯,m. This indicates that there exists L∈N such that

(5) $$(f_{i(kn_{\ell }+r)+1}^{i(k-r)}\left(\hat{x}\right),f^{i(k-r)}\left(\hat{x}\right))\in D$$

hold for all ℓ≥L and all $\hat{x}\in S$. By using (4), we get that there exist $u_{i}\in U_{i}$ such that

$$f_{1}^{i(kn_{L}+r)}\left(u_{i}\right)\in V_{i},\;i=1,2,\ldots ,m.$$

Then by (3), we have

(6) $$(f^{i(k-r)}\left(p_{i}\right),f^{i(k-r)}\left(f_{1}^{i(kn_{L}+r)}\left(u_{i}\right)\right))\in D,\;i=1,2,\cdots ,m.$$

According to (5)

(7) $$(f_{i(kn_{L}+r)+1}^{i(k-r)}\left(f_{1}^{i(kn_{L}+r)}\left(u_{i}\right)\right),f^{i(k-r)}\left(f_{1}^{i(kn_{L}+r)}\left(u_{i}\right)\right))\in D$$

By (6) and (7)

$$(f_{i(kn_{L}+r)+1}^{i(k-r)}\left(f_{1}^{i(kn_{L}+r)}\left(u_{i}\right)\right),f^{i(k-r)}\left(p_{i}\right))\in D\circ D.$$

$$i.e.,\;(f_{1}^{ik(n_{L}+1)}\left(u_{i}\right),f^{i(k-r)}\left(p_{i}\right))\in D\circ D,\;i=1,2,\ldots ,m.$$

According to the hyperthesis, $(x_{i},p_{i}^{\prime })\in D$ and $(x_{i},f^{i(k-r)}\left(p_{i}\right))\notin E$. Since $p_{i}^{\prime }=f_{1}^{i(n_{L}+1)k}\left(p_{i}^{\prime }\right)$, then $(x_{i},f_{1}^{i(n_{L}+1)k}\left(p_{i}^{\prime }\right))\in D$. Therefore

$$(f_{1}^{i(n_{L}+1)k}\left(p_{i}^{\prime }\right),f_{1}^{i(n_{L}+1)k}\left(u_{i}\right))\notin D,\;i=1,2,\cdots ,m.$$

Hence (S,U) is N-sensitive. □

## 5. Examples

In this section, examples will be given to support the Theorems.

**Example** **1.** *Let I be the unit interval [0,1], consider a nonautonomous dynamical system $\left(I,f_{1,\infty }\right)$, the maps $f_{n}\left(n\geq 1\right)$ are defined by*

$$f_{1}\left(x\right)=x,x\in \left[0,1\right],$$

(8) $$f_{n}\left(x\right)=\left\{\begin{array}{cc} \frac{8}{3}x & x\in [0,\frac{3}{8}]\\ -\frac{8}{3}x+2 & x\in [\frac{3}{8},\frac{3}{4}]\\ 4x-3 & x\in [\frac{3}{4},1] \end{array}\right.n\geq 2$$

Denote that $f=f_{n}\left(x\right)\left(n\geq 2\right)$. As *f* is triangle-tent map, the values of $f^{n}$ alternate between 0 and 1 in the images. For any nonempty open set U⊂[0,1], there is a large enough n∈N such that [0,1] is covered by $f^{n}\left(U\right)$. Hence *f* is sensitive. Since sensitivity is equivalent cofinite sensitivity for continuous map in the interval, then *f* is cofinitely sensitive. Therefore $\left(\left[0,1\right],f_{1,\infty }\right)$ is cofinitely sensitive. It follows that $\left(\left[0,1\right],f_{1,\infty }\right)$ is strongly multi-sensitive and N-sensitive.

**Figure 1:**
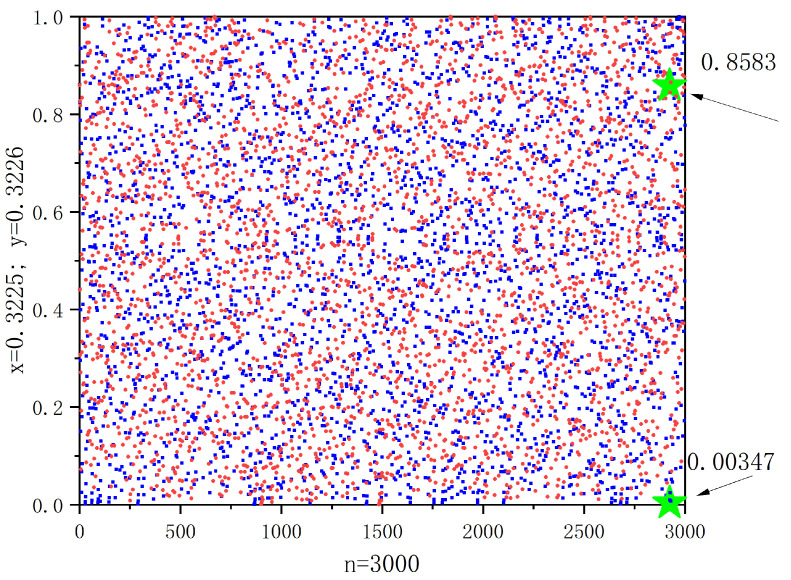
Chaotic behaviors of $f_{1,\infty }$ in Example 1 with the initial date x=0.3225, y=0.3226 and n=3000.

Figure 1 shows a computer simulation with an explanation of sensitivity. As can be seen in the Figure 1, the orbit of *x* (or *y*) is ergodic and disordered. The initial values x=0.3225 and y=0.3226 iterate for 3000 times, respectively, as shown by the blue and red dots, respectively. It is evident that there is a significant disparity between the iterations after a certain number of times, even with a tiny variation between the beginning values *x* and *y* ($f_{1}^{2924}\left(x\right)=0.8583,f_{1}^{2924}\left(y\right)=0.0035$).

**Example** **2.** *Let I be the closed unit interval [0,1] and $f_{n}\left(n\geq 1\right)$ be defined by*

(9) $$f_{1}\left(x\right)=\left\{\begin{array}{cc} \frac{8}{5}x & x\in [0,\frac{1}{2}]\\ \frac{4}{5} & x\in [\frac{1}{2},1] \end{array}\right.$$

(10) $$f_{2}\left(x\right)=\left\{\begin{array}{cc} \frac{4}{3}x & x\in [0,\frac{1}{2}]\\ \frac{2}{3} & x\in [\frac{1}{2},1] \end{array}\right.$$

(11) $$f_{n}\left(x\right)=1-\left|1-2x\right|,n\geq 3$$

As each $f_{n}\left(x\right),n\geq 3$ is tent map, $\left(\left[0,1\right],f_{3,\infty }\right)$ is cofinitely sensitive. Denote that $f=f_{n},n\geq 3$. Actually, for any non-empty open set U⊂[0,1]. If 0∈U, since there exists x∈U and m>0 such that $f^{m}\left(x\right)=1/2$, and noting that f(0)=f(1)=0,f(1/2)=1, it follows that $f^{n}\left(U\right)=\left[0,1\right]$ for n=m+1. If 0∉U, let *J* be an open interval of *U*. Suppose that for each m≥0, $\frac{1}{2}\notin f^{m}\left(J\right)$, then the length of $f^{m}\left(J\right)$ is $2^{m}$ times the length of *J*. Since *m* increases to infinity later, there must be some m>0 such that the length of $f^{m}\left(J\right)$ greater than 1. This forms a contradiction. Therefore, there exists $m_{0}>0$ such that $\frac{1}{2}\in f^{m_{0}}\left(J\right)$. Thus, as previously stated, there is also an *n* such that $f^{n}\left(U\right)=\left[0,1\right]$. This implies that ([0,1],f) is sensitive. According to the equivalence between sensitivity and cofinite sensitivity on the interval ([6] Theorem 2), $\left(\left[0,1\right],f_{3,\infty }\right)$ is cofinitely sensitive.

For any non-empty open set $U\subset [\frac{1}{2},1]$, denote $g\left(x\right)=f_{1}\left(x\right)$ and $h\left(x\right)=f_{2}\left(x\right)$, noting that $g^{n}\left(U\right)=\left\{\frac{4}{5}\right\}$ and $h^{n}\left(U\right)=\left\{\frac{2}{3}\right\}$ for any n≥0, thus maps $f_{1}$ and $f_{2}$ are not feebly open. Since $\left\{f_{1}^{n}\left(x\right):x\in \left[\frac{1}{2},1\right],n\geq 2\right\}=\left\{\frac{2}{3}\right\}$, $\left(\left[0,1\right],f_{1,\infty }\right)$ is not even sensitive. This example suggests that the condition “feebly open” is necessary in the converse of Theorem 4.

## 6. Conclusions

This paper introduces the concept of multi-sensitivity with respect to a vector in the context of non-autonomous dynamical systems on uniform spaces, providing an insights into N-sensitivity and strongly multi-sensitivity, as well as their behaviors under varying conditions. Compared to previous work, this article further extends the concept of strongly sensitivity and enriches the research on strongly sensitivity. Existing research work forms the basis for our work, and our work further extends and expands upon this existing research. We acknowledge the findings and methodologies established by previous studies and use them as a starting point. Our work builds upon the foundation laid by previous research and pushes the boundaries by delving deeper into the subject matter or applying the concepts in different contexts. We presents a sufficient condition under which a nonautonomous dynamical system on an infinite Hausdorff uniform space demonstrates N-sensitivity. However, what is a sufficient condition for a system to be strongly multi-sensitive? This is an interesting future direction of work.

## Data Availability

Data will be made available on reasonable request.
